# Cell-Type Specific Oxytocin Gene Expression from AAV Delivered Promoter Deletion Constructs into the Rat Supraoptic Nucleus in vivo

**DOI:** 10.1371/journal.pone.0032085

**Published:** 2012-02-21

**Authors:** Raymond L. Fields, Todd A. Ponzio, Makoto Kawasaki, Harold Gainer

**Affiliations:** Laboratory of Neurochemistry, National Institute of Neurological Disorders and Stroke, National Institutes of Health, Bethesda, Maryland, United States of America; Yale School of Medicine, United States of America

## Abstract

The magnocellular neurons (MCNs) in the hypothalamus selectively express either oxytocin (OXT) or vasopressin (AVP) neuropeptide genes, a property that defines their phenotypes. Here we examine the molecular basis of this selectivity in the OXT MCNs by stereotaxic microinjections of adeno-associated virus (AAV) vectors that contain various OXT gene promoter deletion constructs using EGFP as the reporter into the rat supraoptic nucleus (SON). Two weeks following injection of the AAVs, immunohistochemical assays of EGFP expression from these constructs were done to determine whether the EGFP reporter co-localizes with either the OXT- or AVP-immunoreactivity in the MCNs. The results show that the key elements in the OT gene promoter that regulate the cell-type specific expression the SON are located −216 to −100 bp upstream of the transcription start site. We hypothesize that within this 116 bp domain a repressor exists that inhibits expression specifically in AVP MCNs, thereby leading to the cell-type specific expression of the OXT gene only in the OXT MCNs.

## Introduction

It is widely accepted that cell-type specific gene expression determines cell identity in the nervous system [1], [2] and that specific neuropeptide expression is a robust marker that can distinguish between neuronal phenotypes [3], [4]. The neurohypophysial peptide hormones, oxytocin (OXT) and vasopressin (AVP), are well known for their roles in the periphery to stimulate milk let-down during lactation and induce uterine contractions during parturition, and to regulate systemic salt and water balance, respectively [5], [6], [7]. In recent years, however, these neuropeptides have been shown to be released within the central nervous system and to also have important physiological and behavioral functions [7], [8], [9], [10], [11]. The OXT and AVP neuropeptides are predominantly synthesized in three populations of magnocellular neurons (MCNs) in the hypothalamo-neurohyophysial system (HNS) of the brain, consisting of the supraoptic, paraventricular, and accessory nuclei. The rodent supraoptic nucleus (SON) is unique in that it contains only one neuronal phenotype, the MCN, which is primarily represented by two distinct MCN subtypes in approximately equal numbers, that are distinguished by their respective commitments to express either the OXT or the AVP gene [12]. These selectively expressing MCNs account for about 97% of the. MCN population in the SON, and ∼3% of the MCNs have been shown to coexpress both peptides [13], [14], [15], [16].

The genes for the OXT and AVP peptides are very similar, in that both contain 3 exons and 2 introns and are located on the same chromosome. They are separated from each other by as little as 3.5 kb in the mouse and 11 KB in the rat and are oriented in a tail to tail fashion, and therefore are transcribed towards each other from opposite strands of the DNA [6]. An unanswered, central question in this field has been what mechanisms are responsible for the highly selective regulation of the cell-type specific expression of OXT and AVP genes in the MCNs of the SON. In attempts to address this question, many molecular studies on the regulation of the expression of these genes have been done using heterologous cell lines to determine which cis-elements in the OXT and AVP genes are responsible for the selective MCN expression. (reviewed in Burbach et al [6]). The data obtained from these models have been generally useful, but it is clear that heterologous cell lines, because of their de-differentiated states, are not appropriate models for the study of the cell-type specific regulation of the highly differentiated *in vivo* phenotypes. One experimental approach that investigators have used to address this question of cell-type specific expression of the OXT and AVP genes has been the use of transgenes containing various combinations of the OXT and/or AVP gene sequences in transgenic rodents [17]. The data from these studies identified a region <0.6 kbp upstream of OXT exon I as having all of the necessary components for cell-type specific expression of the OXT phenotype in the SON [18], [19].

In this paper, we make use of a new experimental approach to perform promoter deletion analysis *in vivo*, that is to use stereotaxic injections of viral vectors for gene transfer into the SON in order to further dissect the cis-elements in the ∼0.6 kbp domain upstream of the OXT gene transcription start site that regulate the cell-type specific expression. This *in-vivo* method uses AAV vectors expressing OXT-promoter deletion constructs and utilizes the enhanced green fluorescent protein (EGFP) as the reporter. The AAV constructs are stereotaxically injected into the rat brain above the SON and 2 weeks post injection the rats are sacrificed and assayed by immunohistochemistry for co-localization of EGFP expression within OXT or AVP MCNs. Using this method we have been able to identify an approximately 116 bp region upstream of the transcription start site (TSS) in the OXT gene promoter which is responsible for conferring the selectivity of OXT gene expression in the SON.

## Materials and Methods

### Animals

Adult male Spraque-Dawley rats were obtained from Charles River Laboratories (Wilmington, MA) and maintained under normal laboratory conditions (temperature: 21–23°C, 12 hour light-dark cycles with lights on at 6:00 AM) with access to unlimited food and drinking water. Rats were caged individually following surgical procedures. All animal procedures using rats were carried out in accordance with National Institutes of Health (NIH) guidelines on the care and use of animals and an animal study protocol approved by the National Institute of Neurological Disorders and Stroke (NINDS) Animal Care and Use Committee.

### Plasmid Constructs

**Figure 1:**
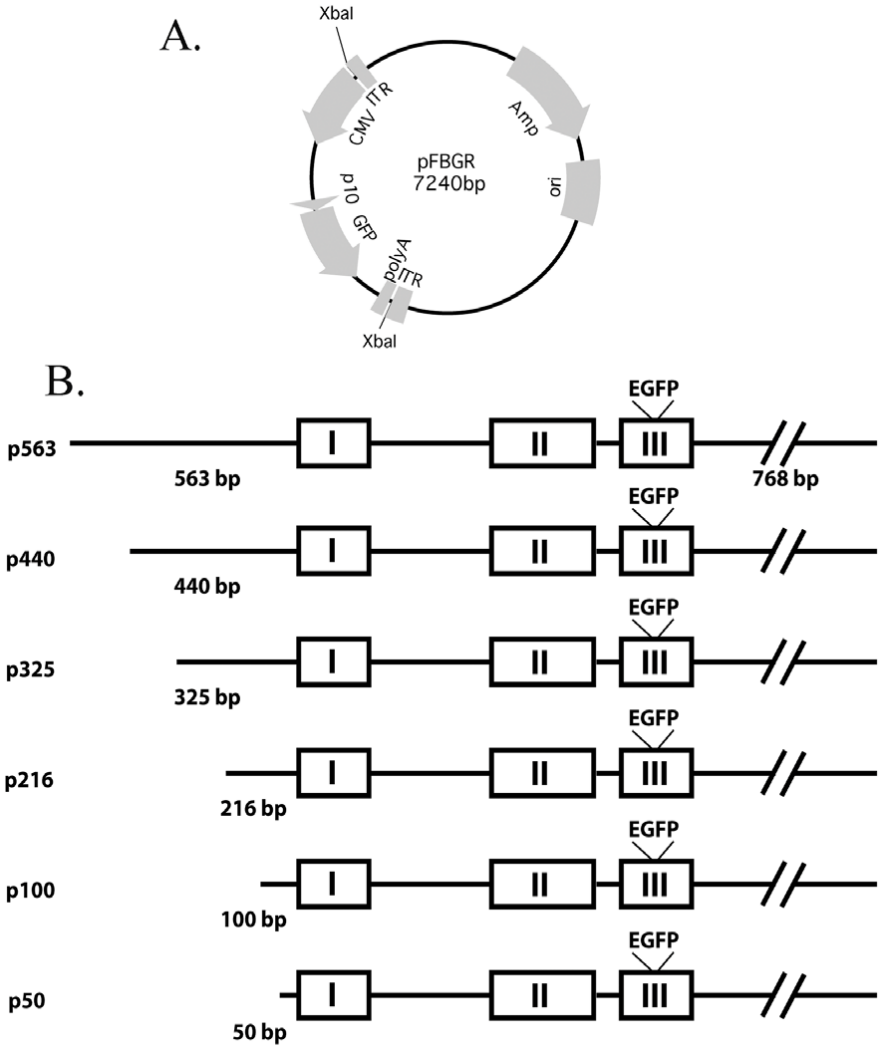
Description of AAVs containing OXT promoter deletion constructs used in this study. A. Shows plasmid pFBGR, which was used for making all constructs, this plasmid contains the AAV left and right inverted terminal repeats (ITR). B. shows diagrams of constructs made containing sequences 568, 440, 325, 216, 100, and 50 bp upstream of the transcription start site of the OXT gene. These constructs contained all the three exons and two introns in the OXT gene and with the EGFP reporter inserted at the end of the coding region of exon III, followed by 768 bp downstream of OXT exon III. All constructs were placed into pFBGR between the XbaI sites after removal of the 2291 bp XbaI band (see panel A).

**Figure 2:**
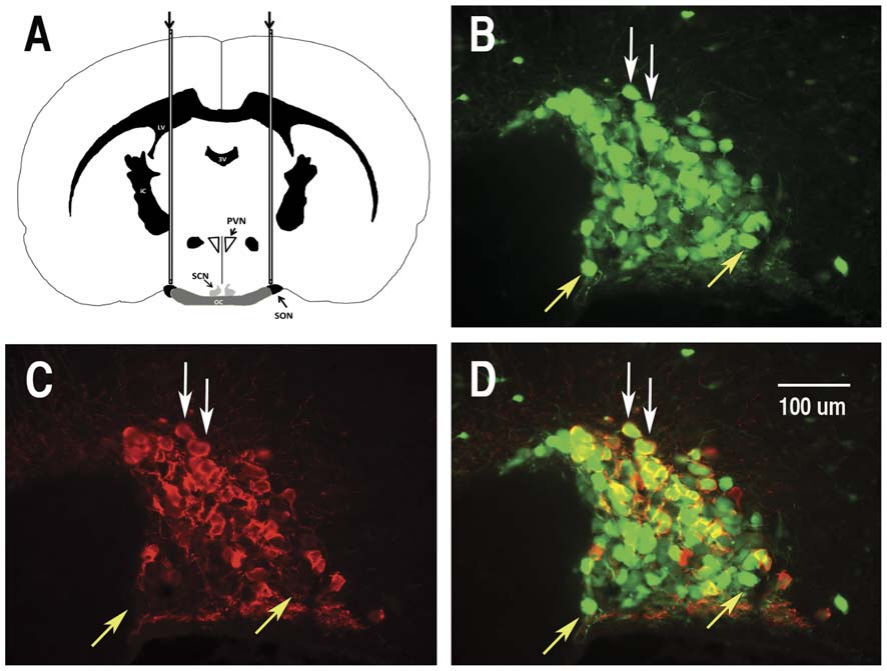
Efficacy of targeting both OXT and AVP magnocellular neurons (MCNs) in rat supraoptic nucleus (SON) by stereotaxic injections of AAVs. The AAVs used were chimeric AAV6 serotypes with an AAV2 ITR and an AAV6 capsid (see Methods). A. Illustrates a rat brain coronal section showing the placement of 30 gauge needles used for bilateral injections of AAVs to target the SON. Stereotaxic coordinates are presented in Methods. Panels B–D show the results of injecting an AAV containing the CMV promoter fused to an EGFP reporter into the rat SON. B. Illustrates EGFP fluorescence in the MCNs of the SON found two weeks after injection. C. shows OXT MCNs in the SON after staining with PS 38 antibody (an OXT MCN marker). D. Shows merged EGFP fluorescence and PS 38 staining. White Arrows in panels B–D show MCNs in the SON that express EGFP co-localized with PS 38-immunoreactivity. Yellow arrow in panels B–D show MCNs that express EGFP which is not co-localized with PS 38-immunoreactivity, and hence are presumptive AVP MCNs. Abbreviations in A: cc, corpus callosum; LV, lateral ventricle; f, fornix; 3 V, third ventricle; ic, internal capsule; PVN, paraventricular nucleus; SCN, suprachiasmatic nucleus; SON, supraoptic nucleus; OC, optic chiasm. Scale line in D is the same for B and C.

The pFBGR plasmid shown in Fig. 1A (obtained from Dr. Robert Kotin, NIH\NHLBI) was used to construct all the AAVOXT plasmids. pFBGR contains the AAV inverted terminal repeats, ITRs, flanking the CMV promoter and EGFP reporter. Bacterial transposon Tn7 L and R attachment sites are located outside of the ITRs allowing the constructs to be incorporated into the bacmid bMON14272 (Life Technologies, Carlsbad, CA USA). The CMV-EGFP expressing AAV used in Fig. 2 B–D was made using pFBGR as the starting plasmid. The OXT promoter constructs to be inserted in the AAV vector (Fig. 1B) were made by digesting pFBGR with XbaI to remove the sequences in between the ITRs and ligating the OXT constructs into the XbaI sites. XbaI sites are directly internal to both ITR sequences in the pFBGR plasmid. All OXT promoter inserts were made using PCR primers, with XbaI sites at the 5′ends, to amplify the construct of interest out of the plasmid OXTIII.EGFP.IGR3.6 previously described in Fields et al [20]. The pFBOT563 construct was made using p563F GCTCTAGAGCGTCTACACAGCAGGTTCTAATACAGA and p563R GCTCTAGAGCCATGCCCTCACTTCTGCCCATTACT primers to amplify a 3 kb band out of the plasmid OXTIII.EGFP.IGR3.6. This band contains 563 bp of the OXT 5′UTR, OXT exons I–III with EGFP inserted at the end of the OXT coding sequence of exon III followed by 768 bp of the sequence downstream of OT exon III. The 3 kb band was ligated into pFBGR that was digested with XbaI. pFBOT440, 325, 216 and 100 were made by using a forward PCR primers (TCTAGATTTTATTTTAATTTGGTCTGTTAACTCTG, TCTAGAGCCCTATCCTGCCTTATTCTGAG, TCTAGAACCCCTTCCAGGCTGCTTC, and TCTAGACACCCAAGAGACCTTCTGTGACC respectively) to reduce the length of the OT 5′UTR and a reverse primer, OTR, (CGCTAAAGGTATTCCCAGAAAGTGG) that binds to exon III of pFBOT563 downstream of a NotI site in pFBOT563 used in pFBOT563 as a linker to clone in the EGFP reporter. All of the 440-100 forward primers have an Xba I site at 5′ end of the primer. The 440-100 PCR products were digested with XbaI and NotI and then cloned into pFB563 digested with NotI and the XbaI site upstream of the OXT 5′ UTR(via partial digestion). This gives plasmids with 563, 440, 325, 216, and 100 bp of the OXT 5′UTR as shown in figure 1B. The pFBOT50 construct was made using PCR primers OT50F GATCGAGCTCTGCTCCACCATGGCAGTGCCA and OT50R GATCGGATCCCGGGCCGCAACTCCGA to PCR out a 1.8 kb band from OXTIII.EGFP.IGR3.6. This band was digested with SacI and BamHI and ligated into SacI BamHI digested pFBOT100 giving a plasmid similar to pFBOT100 but with only 50 bp of the 5′ UTR. All of the above PCR reactions were done using the Expand High Fidelity PCR System (Roche, Germany) according to the manufacturers instructions with a thermal cycler program of 94°C 2 min1×, 94°C 15 sec 55°C 30 sec 72°C 1.5 min 10×, 94°C 15 sec 55°C 30 sec 72°C 1.5 min plus 5 sec per cycle 25×, 72°C 7 min 1×.. All of the OXT-promoter deletion constructs made for this study are shown in Fig. 1B.

### Baculovirus and AAV construction

We used a recombinant Baculovirus (rBV) method that employed insect cells for the production of AAVs which results in an increased AAV production/transfected cell as compared to the method using 293T cells [21]. rBVs were made as precursors to AAV production in Sf9 insect cells using the Bac-to-Bac Baculovirus Expression System, (Life Technologies). In order to calculate accurate rBV ratios and Multiplicity of Infections (MOIs) during the production of the AAVs, rBV titers were determined by QPCR using a procedure modified from Lo et. al [22]. In this method we used dilutions of intact rBVs as the template for QPCR along with primers targeting the BV genome. Primers were designed using the MacVector 7.0 program (MacVector, NC, USA) and the *Autographa californica* multicapsid nucleopolyhedrovirus DNA sequence (Accession# NC_001623). The primer sequences used were B.V.109103–109124 5′- ATGAAGCACAGTGTCCACGGTC -3′ and B.V. 109286-109264 5′- GGTGAAGCGGCAGAATAACAATC -3′ which amplify a 184 bp amplicon from the viral envelope glycoprotein. The QPCR reaction was performed in a Smart Cycler RT-PCR system (Cepheid, CA, USA). The reactions were done using Real-Time SYBR Green PCR master mix (SA Biosciences, MD, USA) according to the manufacturer's protocol with 1 ul of diluted template (1∶1000 or 1∶10,000) added. The run profile was 95°C for 15 min 1×, 95°C for 15 sec, 60°C for 30 sec, 72°C for 30 sec 40×, followed by a 60°C–95°C melt curve. To generate a standard curve for rBV tittering, DNA was isolated from rBV Bac-RepCap6 (a gift from Dr. Robert Kotin, NIH\NHLBI) according to the method of O'Reilly et al [23], and serial dilutions of the rBV Bac-RepCap6 DNA were used as control template. Titers for all newly manufactured rBV stocks were determined from this standard curve.

In preliminary experiments, we found that of all the serotypes tested (AAV2, AAV5, AAV6, AAV8, and AAV9) the AAV 5 and 6 serotypes were the most efficient in transducing the MCNs in the SON. A rBV containing the AAV2 rep genes and the AAV6 capsid genes, Bac-RepCap6, which produces a serotype 6 AAV was used for the AAV packaging reactions [24] rBVs containing the OXT constructs shown in Fig. 1B were combined with the Bac-RepCap6 rBV at a ratio of 3∶1 and used to infect 200 ml of Sf9 cells at density of 2×10^6^ cells/ml in suspension culture at a MOI of 3 according to the method of Negrete et al [25]. Cells were incubated for 3–4 days at 27°C while shaking at 115 rpm until lysis-induced mortality reached at least 50% as measured by hemocytometer counting of Trypan Blue stained cells. Cells were harvested by centrifugation at an RCF of 2000 g for 10 min and the supernatant was saved. The cell pellet was resuspended into 10 ml of sterile PBS, and the cells subjected to three freeze/thaw cycles and centrifuged again at 2000 g for 10 min. The supernatant from this spin was combined with the supernatant from the first spin. MgCl_2_ was added to a final concentration of 2 mM and the mixture incubated at 37°C for 30 min with 20 U/ml benzonase (Sigma). After this incubation, PEG -8000 (Sigma, St. Louis,MO) was added to a final concentration of 2% and the mixture incubated over night at 4°C. The solution was centrifuged at RFC 4000 g for 30 minutes to pellet the viral particles. The supernatant was then discarded and the viral pellet re-suspended into 12 ml of CsCl with a refractive index of 1.372. Recombinant AAV was purified through a CsCl gradient by centrifuging at 38,000 rpm in a SW Ti-41 rotor (Beckman Coulter, Brea, CA) for 48 hrs. Fractions with a refractive index between 1.378-1.368 were pooled and concentrated, while exchanging the buffer to 2 mM MgCl_2_ in PBS, using a microcon YM-100 spin filter (Millipore, MA).

QPCR was performed on the purified and concentrated AAVs to determine their titers. The AAV titer was determined by comparison with a standard curve made using pFBOT100 plasmid DNA and used the following primers: EGFP F-ACCCTCGTGACCACCCTGAC and EGFP R-ACCTTGATGCCGTTCTTCTGC which amplify a 130 bp fragment of the EGFP gene. PCR conditions were the same as above for the rBV titers. AAV titers are expressed as viral genome/ml (vg/ml). Viral titers used in all experiments ranged between 1–7×10^12^ vg/ml as determined by the above QPCR method

### Stereotaxic targeting of AAVs into the rat SON

2–3 month old rats (270–425 g) were anesthetized with 5% isoflurane (Baxter, Deerfield, IL) in a Stoelting gas anesthesia adaptor for stereotaxic instruments (David Kopf Instruments, Tujuna, CA) and placed into a stereotaxic apparatus (Stoelting, Wood Dale, IL) in flat skull position. The scalp of the rat was shaved and then sterilized with betadine, 30% betadine: 70% ethanol, and lastly 70% ethanol. A rostral-caudal incision was made to access the skull and two holes were drilled dorsal to the SONs at coordinates 1.3 mm posterior to bregma; 1.8 mm medial lateral on each side as determined using a stereotaxic atlas [26]. A 10 ul syringe with a 30 gauge needle was placed −8.8 to −9.0 mm ventral to bregma and 3 ul of the AAV viral constructs was delivered to the SON area at a rate of 0.3 ul/min. Viral titers used in the experiments ranged between 1–7×10^12^ vg/ml. Following the injections, the holes were filled with bone wax (Medline, IL) and the incision closed with interrupted sutures. Ketoprofen (Fort Dodge Animal Health, IA) was administered (5 mg/kg) after surgeries intraperitoneally. Fig. 2A shows a coronal section of a rat brain in a diagram illustrating the method used for injections.

### Salt-loading of rats

In some experiments, systemic hyperosmotic stimulation of rats was done. For this purpose, rats were salt-loaded by being given 2% NaCl in their drinking water for 1 week prior to being sacrificed at two weeks post injection of the AAV constructs.

### Immunohistochemistry (IHC)

Rats were killed 2 weeks after injection by overdose of isoflurane anesthesia (Baxter, Deerfield, IL). Immediately upon death, rats were perfused transcardially with 50–100 ml of phosphate-buffered saline (PBS) followed by 200–250 ml of fixative solution (4% paraformaldehyde in PBS, pH 7.4) at a perfusion rate of 5 ml per min. Following the perfusion, the brains were removed and cryoprotected with 5% sucrose, 0.9% saline for 4 hr to overnight at 4°C, followed by 10% and 15% sucrose, 0.9% saline in a similar manner and then stored at −80°C until use. Coronal sections (16 um) were cut on a cryostat (Reichert-Jung 2800; Frigocut, Heidelberg, Germany) and mounted onto colorfrost slides (12-550-19, Fisher Scientific, Pittsburgh, PA). The slides were incubated in fixative solution for 5 min then washed 3× in PBS and incubated in PBS containing 0.3% Triton-X for 5 min, washed 3× in PBS followed by incubation in PBS containing 10% NGS 0.6% triton x-100 as blocking agent for 30 min and rinsed 3× in PBS. Brain sections were incubated with the mouse monoclonal antibodies PS38 against OXT-neurophysin (OXT-NP), PS41 against AVP-NP or PS 45 a pan-specific NP antibody at dilutions of 1∶200 in PBS with 1%BSA [27], [28], or a polyclonal antibody against the GFP protein, Ab-2090, (Abcam, Cambridge, UK) used at a dilution of 1∶1000 and incubated overnight at 4°C. After incubation in primary antibodies, the slides were rinsed three times for 5 min in PBS at room temperature. The AVP-IR and OXT-IR slides were incubated in Alexa Flour® 594-conjugated goat anti-mouse (Molecular Probes, Eugene, OR) secondary antibody (1∶500) to yield cytoplasmic red fluorescence. Alexa Flour® 488(Molecular Probes) secondary antibody was used to yield cytoplasmic green fluorescence in the experiments using antibodies to EGFP. After immunohistochemistry the slides were air dried in the dark then hydrated with a few drops of dH_2_O. Cover slips were placed over them and images taken using an Eclipse E400 fluorescent microscope (Nikon, Melville, NY) equipped with a Exi Fast (QImaging, BC, Canada) CCD camera. All the immunohistochemical data shown are representative of 3–5 independent experiments on rats. Measurements of the numbers of either AVP- or OXT-identified MCNs that showed colocalization of EGFP expressed from the AAV vectors injected into the SON, were made by visual superimposition and cell-to-cell matching of the EGFP stained and specific NP antibody stained cells after double-label immunostaining.

## Results

### Efficacy of AAV vector-mediated gene transfer into OXT and AVP MCNs in the rat SON

The evaluation of the cell-type specific expression of any gene construct delivered into the SON by stereotaxic microinjection (Fig. 2A) depends on the equivalent effectiveness of the AAV vector used to transduce both the OXT and AVP MCNs by this method. Figure S1 shows the intense labeling of the SON after injection of an AAV containing a CMV-EGFP construct, and the expression of EGFP in various non-MCNs surrounding the SON where the virus could reach. Most important, we determined whether such injections of AAV-6 containing CMV-EGFP constructs into the SONs of rat brain would succeed in transducing both populations of MCNs. It should be noted here that the AAV-6 vector is not known to show neuronal specificity with respect to its transduction efficacy [29], [30], [31], and hence would be expected to transduce both MCN phenotypes equivalently. Figs. 2B–D show the results of such an experiment in which an AAV expressing the EGFP reporter under the control of a CMV promoter was injected into the SON. Fig. 2B shows that two weeks after injection the EGFP green fluorescence fills all the MCNs in the SON, thus showing that this injection procedure effectively transduces all of the MCNs in the SON. Fig. 2C identifies the OXT MCNs in the same section stained red using the PS38 antibody (the OXT-MCN marker). Fig. 2D shows a merge of the images in fig. 2B and 2C. The white arrows in Fig. 2B–D depict the oxytocin cells in the SON that clearly coexpress EGFP, and the yellow arrows show EGFP expression in cells that clearly do not express oxytocin, and are presumed to represent the vasopressin MCNs, since OXT and AVP MCNs are the only neuronal phenotypes found in the SON [12]. In a separate set of experiments we injected the AAV-CMV-EGFP constructs into the SON and determined the efficiency of the AAV-6 to transduce all the MCNs in the SON by comparing the MCNs expressing EGFP to MCNs that were double labeled with the pan-specific neurophysin monoclonal antibody, PS 45, that immunostains all the MCNs in the SON (see Fig. S2). After evaluating 2,499 EGFP labeled cells in 9 injected SONs from 5 rats, we found that the EGFP expressing cells represented 69.1%±29.5 (SD) of the total MCNs in the injected SON, and of these 97.7%±4.8 (SD) were double labeled by PS 45. Therefore, we conclude that the efficiency of transduction with a CMV promoter is about 69% and that both the OXT and AVP MCN phenotypes are transduced by the AAV vector that is stereotaxically injected in this manner (see Fig. S2).

### Validation of the use of AAV gene transfer for evaluating cell-type specific gene expression in the SON in vivo

**Figure 3:**
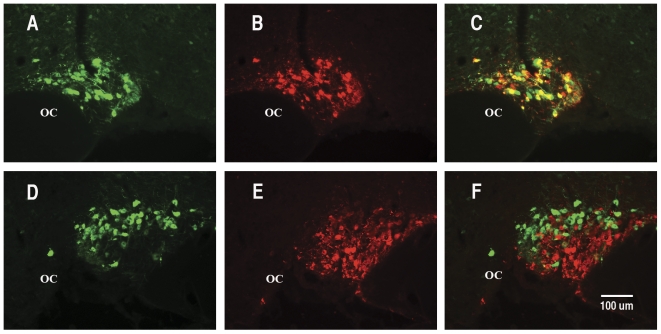
Specificity of EGFP expression from the p563 AAV construct. Immunohistochemistry was performed two weeks after injection of the AAVs into the rat SON. Brain sections in A and D show EGFP fluorescence, in B, PS38 antibody staining, in C, merged EGFP fluorescence and PS38 staining, in E, PS41 antibody staining, and in F, merged EGFP fluorescence and PS41 staining. Note that while the EGFP is abundantly expressed in the OXT MCNs (panel C), there is virtually no expression of the EGFP in the AVP MCNs (panel F). Abbreviations: OC, optic chiasm. Scale line in F is the same for all panels.

Given the above demonstration that both OXT and AVP MCNs in the SON are effectively transduced by the injected AAV vector, we then tested an AAV that contained 563 bp of the OXT promoter followed by the complete OXT gene with the EGFP reporter placed at the end of the exon III coding region and included 768 bp of the untranslated region downstream of exon III (see p563 in Fig. 1B). Since this 563 bp OXT promoter had previously been shown to produce cell-type specific expression of a reporter in both transgenic mice [18] and in organotypic culture [20] experiments, this AAV also served as a positive control for our strategy to use AAV as a means to study the ability of various promoter deletions to produce cell-type specific expression in the MCNs of the SON in vivo. Fig. 3 shows the EGFP expression patterns in the rat SON that are typically observed two weeks after injecting this AAV into to the SON. In preliminary experiments we determined that waiting two weeks after injection of the AAVs was sufficient time to clearly observe the expressed EGFP in the SON by immunofluorescent IHC (data not shown), Consequently, unless otherwise stated, we routinely determined expression of the EGFP reporter by immunofluorescence assays two weeks post injection.

Figs. 3A–C illustrate a brain section with the EGFP fluorescence shown in panel A, PS 38 antibody (OXT cell marker) staining in panel B, and the merge in panel C. The yellow cells in the merged view in panel C show that the EGFP expression is clearly occurring in many OXT MCNs in the SON. However, there are also green cells that appear to not to colocalize with the OXT marker. This apparent lack of colocalization is due to the fact that cells that have more intense green fluorescence than their colocalized red fluorescence appear green in these unmanipulated photographs. Therefore, it is difficult to illustrate the full extent of colocalization by looking for exclusively yellow and no green cells in these untouched photographs. However, the lack of colocalization of the EGFP and the red AVP specific MCN marker in these photographs, which is not influenced by this photographic artifact, provides a more definitive test of cell-type specific gene expression. Such a test is illustrated in Figs. 3D–F which shows another brain section from the same rat SON with the green fluorescence in panel D, and the PS 41 antibody (AVP cell marker) staining red in panel E, and the merge is shown in panel F. In this case, the merged view in panel F shows that the green fluorescence (EGFP expression) is clearly occurring in MCNs that do not express the AVP marker in red (presumably these are OXT MCNs). In order to get a quantitative assessment of these colocalizations, in separate experiments we injected the p563 OXT-promoter-EGFP AAV into the SON and double label immunostained with either PS 38, an OXT–specific or PS 41 an AVP-specific neurophysin antibody. We then systematically evaluated the EGFP expressing cells in the SON and compared them to their colocalized PS-38 or PS41 immunoreactivities as described in Methods. The results showed that of 938 EGFP labeled cells evaluated in 6 injected SONs from 4 rats, we found that the EGFP was 95.0%±3.3 (SD) colocalized in identified (PS 38-ir) OXT MCNs, whereas of 1898 EGFP labeled MCNs counted in 6 SONs from 4 rats, we found that the EGFP expressing cells were only 3.7%±2.2 (SD) colocalized in identified (PS 41-ir) AVP MCNs. These quantitative data clearly correspond to our conclusions about specificity which were drawn from our interpretations of the images shown in Figure 3. The data in Fig. 3D–F are also consistent with the well known observation that OXT MCNs in the SON are located dorsal to the AVP MCNs at this anatomical level of the nucleus. Therefore, we conclude that there is virtually no expression of the p563 OXT promoter construct in the AVP MCNs in the SON, a finding consistent with our previous transgenic mouse data [18]. Also similar to the transgenic findings, the p563 OXT promoter construct shown in Fig. 1 did not cause ectopic expression, for example it did not produce EGFP expression when injected into cortex (data not shown).

### Promoter deletion analysis of cell-type specific OXT gene expression in the SON

**Figure 4:**
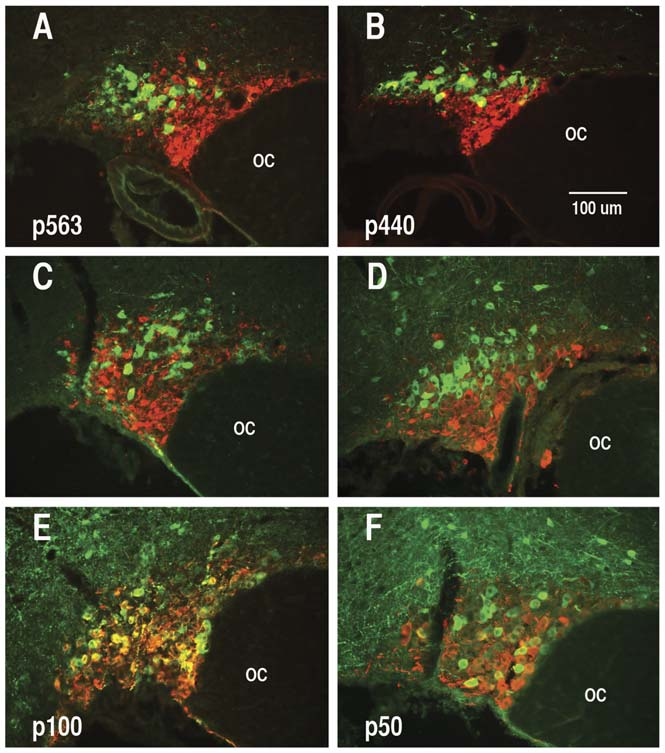
Analysis of the specificity of expression of EGFP from AAVs containing various promoter deletion constructs (shown in **Fig. 1B**) two weeks after their injection into rat SONs. The specific promoter lengths that were injected into the SONs in each of the experiments is shown in the lower left of the panels. Immunohistochemical analyses show that each of the oxytocin promoter AAV constructs was selectively expressed in oxytocin cells. Each panel illustrates merged images of AVP-neurophysin (PS 41) immunoreactivity shown as red, and the EGFF expression shown as green. Note that while the EGFP is clearly not expressed in the AVP MCNs in panels A–D where the promoter constructs contain 216 bp or more of the upstream region, the p100 construct (panel E) and p50 construct (Panel F) shows EGFP expression in both OXT. Hence the key elements regulating of the selectivity of OXT gene expression must reside between in the −216 to −100 bp domain in the promoter. Abbreviations: OC, optic chiasm. The 100 µm scale line in lower right is the same for all panels.

In order to determine the locations of the regulatory elements in the OXT gene that are responsible for its cell specific expression, we injected AAVs that contained the six promoter deletion constructs illustrated in Fig. 1B into rat SONs. These started with the 563 bp construct discussed above and were systematically decreased in length to −50 bp upstream of the TSS (Fig. 1). Fig. 4 summarizes the results of these experiments. The merged views shown in this figure compares the cell locations of EGFP fluorescence to AVP-neurophysin (PS 41) immunoreactivity which represents the definitive test of specificity, and clearly show that the EGFP expression is excluded from the AVP MCNs for all of the constructs, except for the p100 and p50 constructs where the EGFP is expressed in both AVP and OXT MCNs. From these data we conclude that the cell-type specific regulatory element in the OXT promoter is located between −100 and −216 bp upstream of the transcription start site in the OXT gene, and suggests that this domain most likely contains a repressor element in the OXT promoter that suppresses OXT gene expression specifically in the AVP MCNs. The data in Fig. 4 also shows that there are small, unidentified neurons dorsal to the the SON which appear to express EGFP from the p50 to p216 constructs, but these small neurons do not express significant EGFP from the p325 to p563 promoter constructs. Therefore, we propose that there is another repressor element between −216 bp to −325 bp upstream of the transcription start site in the OXT gene that specifically prevents expression in these unidentified dorsal neurons.

**Figure 5:**
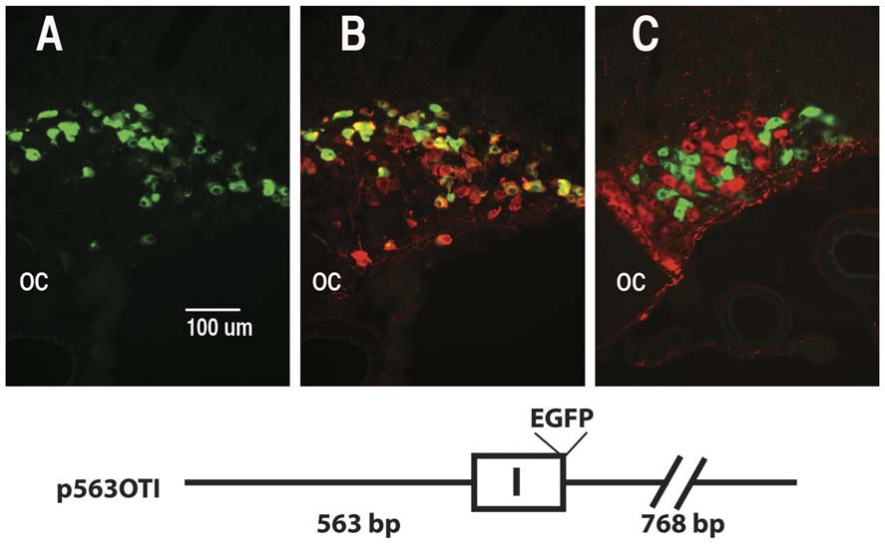
Removal of exons 2 and 3, and both introns does not alter the specificity of expression properties of the OXT promoter. An AAV was made containing the p563 OXT promoter- EGFP reporter construct (pOTI) shown at the bottom of the figure. The construct contains 568 bp of the OXT upstream promoter region followed by OXT exon I with the EGFP reporter gene fused to the end of exon I followed by 768 bp of the region downstream of OXT exon III. The pOXTI AAV was injected into the SON of a salt loaded rat. A. shows EGFP Fluorescence, B. shows a merge of EGFP fluorescence and OXT antibody staining in the same section, C. shows EGFP fluorescence and AVP-NP antibody staining in a different section from the same rat SON. OC = optic chiasm. The scale line in A is the same for B and C.

The constructs used in the previous transgenic studies and the promoter constructs shown in Fig. 1 all contained the intron and exon sequences in the OXT gene. In order to determine whether these regions were involved in the regulation of cell-type specific gene expression in the SON, we injected an AAV vector that had 563 bp of the OXT promoter followed by exon 1 with the EGFP reporter placed at the end of the exon 1 followed by 768 bp of the untranslated region downstream of exon 3 (see the pOTI construct shown in Fig. 5). The results of injecting this construct into the SON are shown in Fig. 5A–C. Panel 5A shows the EGPF fluorescence alone, panel 5B shows the merge of the green EGFP fluorescence with the red PS38 (OXT) antibody stain, and panel 5C shows the merge of the EGFP fluorescence with the red PS41 (AVP) antibody stain. From these data we can see that the expression of EGFP is not found in the AVP cells of the rat SON indicating that removal of both introns and exon 2 and 3 do not alter the cell-type specific expression properties of the OXT promoter in the SON. In this regard, it has recently been shown that the removal of all of the exons, introns, and the 768 bp of the untranslated region downstream of exon 3 from a 2.6 kbp OXT promoter fused directly to EGFP and inserted into an AAV which was then injected into the SON, similarly showed that the EGFP expression was OXT cell-type specific [32].

**Figure 6:**
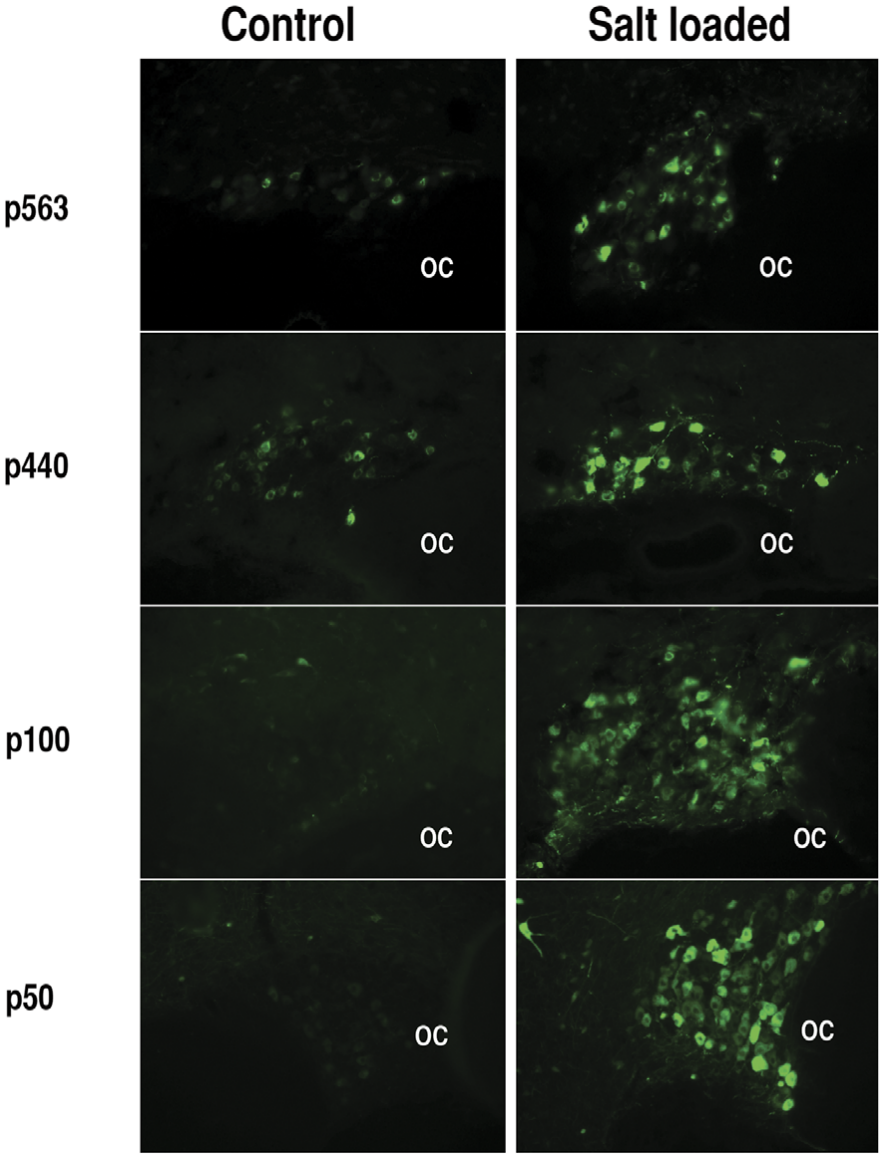
Effects of systemic salt loading on the expression of EGFP in MCNs after injection of various OXT promoter deletion constructs into the rat SON. After injection of the AAVs into the SONs, the control rats were given water to drink for two weeks, whereas the salt loaded rats were were given water for one week, followed by a second week of access only to water containing 2% NaCl (see Methods). Panels show EGFP fluorescence in SONs of control (left column) and salt loaded (right column) rats for each of the p563, 440 bp, p100, and p50 OXT promoter-containing AAVs. Control and salt loaded image capture times are identical for each construct. Note that for each deletion construct the SONs fom salt loaded rats express much more EGFP than the control SONs, this is also the case for the pOXTI promoter construct. OC = optic chiasm. Scale bar in bottom left panel is same for all panels.

It is noteworthy that the fluorescence shown in Fig. 4 for the p216, p100 and p50AAVs required amplification by EGFP antibody staining to be adequately visualized, whereas the p325bp to 563 bp AAVs did not require amplification. We interpret this to be due to lower levels of expression in the promoter regions that are −216 bp or less. This view is reinforced by the data shown for the controls in Fig. 6 whereas the intrinsic fluorescence from the p440 and p563 AAVs were sufficiently robust to be clearly observed without immunohistochemical amplification. In contrast, the shorter deletion constructs were not visible in these normosmotic rats without IHC amplification. For this reason we suggest there may be an enhancer located −216 to −440 bp upstream of the OXT transcription start site.

### Search for a putative osmotically regulated cis- element in the OXT gene

The expression of the oxytocin gene in rat MCNs is known to be dramatically regulated by changes in chronic, systemic osmolality [6], [33], and we were interested in determining if we could use our promoter constructs to locate an element in the OXT gene promoter that is associated hyperosmotic upregulation of OXT gene expression in the SON. In this experiment, we used a standard salt-loading approach to produce chronic hyperosmolality in rats (see Methods). In these experiments, the AAVs were injected in the SON a week before the salt loading procedure began and the rats were salt-loaded by the addition of 2% NaCl in their drinking water at the beginning of the second week prior to being sacrificed. Control animals were given normal water to drink for the entire two weeks following the AAV injection. Fig. 6 shows the results of control versus salt loading on OXT gene expression using the p50, p100, p440 and p563 series of OXT promoter constructs. The left column shows EGFP fluorescence in the SON of control normosmolar rats. The right column shows EGFP fluorescence in the salt loaded, hyperosmotic rats. These data show that the EGFP expression levels increase in the SONs of all of the salt loaded rats as compared to the SONs of control rats. This suggests that the putative element(s) affected by hyperosmotic conditions are found within the first 50 bp of the OXT promoter region or possibly further downstream in the gene. In this regard, experiments by Knobloch et al, [32] have shown that neither the introns nor exons in the OXT gene were necessary for the osmotically induced increase in OXT gene expression. In view of these data, we suggest that the osmotic regulation of the Oxt gene is occurring within or below the −50 bp upstream region of the OXT TSS in the core promoter.

## Discussion

In this paper we examine an important aspect of the molecular mechanisms that regulate cell-type specific OXT gene expression in the MCNs in the SON, and that is to determine which cis-element domains in the OXT gene promoter are responsible for its cell-type specific regulation. Since there are no homologous cell lines available that can serve as appropriate experimental surrogates for the OXT-expressing MCNs found *in vivo*, we and other investigators have previously addressed this issue by using various experimental models that allow for studies of the differentiated OXT MCNs themselves. Previous studies using transgenic mice, have shown that transgenes containing <600 bp of the OXT gene promoter region upstream of the transcription start site (TSS) and the entire intergenic region (3.6 kbp downstream of exon III) could reliably produce robust expression from this construct only in the OXT MCNs, and none at all in the AVP MCNs [18], [19]. Subsequent studies conducted in vitro, using biolistic transfection of the MCNs in organotypic cultures of hypothalamus, showed that 554 bp upstream of the TSS in the OXT gene was sufficient to produce expression in the OXT MCNs [20]. For this reason we chose to start our deletions at −563 bp upstream of the transcription start site.

The above biolistic experiments were primary directed at testing whether downstream elements, i.e., in the intergenic region (the IGR) were necessary for the cell-type specific expression of the OXT and AVP genes in the hypothalamic cultures, as was predicted by the “IGR hypothesis” [34], [35]. In the Fields et al [20] paper we reported that a 432 bp region downstream of exon III in the OXT gene was necessary for the expression of the OXT gene in the hypothalamic slice culture, and that a 178 bp downstream of exon III in the AVP gene was necessary to produce AVP gene expression in the hypothalamic culture. However, further experiments in our laboratory indicated that neither the 178 bp nor the 432 bp domains in the IGR contained the cis-regulatory elements responsible for the cell-type specific expression. The 178 bp and 432 bp domains appeared to contain similar motifs (see Figure 8 in Fields et al, [20]) and we found that these sequences could replace one another to produce the hypothalamic-specific expression observed in the biolistics experiments (Fields and Gainer, unpublished data). From the latter data, we concluded contrary to the IGR hypothesis that the key cis-elements that were responsible for the cell-type specific expression of the OXT gene were not located downstream, but rather were 554 bp upstream of the transcription start site in the OXT gene. However, since the biolistic studies reaffirmed that the 3′ UTR in the OXT gene was necessary for expression in the hypothalamic organotypic cultures, we continued to use the 3′ UTR in the OXT gene in the construct in this study. While the previous studies using transgenic or biolistic model systems provided valuable insights, neither approach appeared to be sufficiently efficient or optimal for the study of multiple promoter deletion constructs of the OXT and AVP genes. For this reason, we decided to develop and use an alternative approach, the AAV gene transfer method in vivo that is used here and to focus our deletions in the 5′ upstream region of the OXT gene. In this regard, it should be noted here that Knobloch et al [32] recently used the AAV approach to show that all the introns and exons, as well as the 3′ UTR (IGR) in the OXT gene were not required for cell-type specific expression of a 2.0 kbp OXT gene promoter in the SON, thereby providing experimental evidence against the IGR hypothesis. It is unclear at present why the findings about the relevance of the 3′ UTR on OXT gene expression differ in experiments using biolistic transfection in vitro versus AAV gene transfer in vivo, and this issue should be examined further in the future.

Transgenic rodent models present several problems for promoter deletion studies not the least of which is the excessive time and cost of the animal husbandry associated with this approach. In addition, the random insertion of the transgene often leads to artifacts such as insertional mutations, and ectopic expression due to enhancer trapping, and the latter problem is increased when the construct is systematically shortened by the process of promoter deletion. The biolistic transfection of organotypic cultures is limited by the very low probability that the gold particles coated with plasmids that are being randomly “shot” at the cultured slice explants will land in the cell nucleus of an OXT-MCN without damaging the cell. Furthermore, in the biolistic transfection method the particles do not penetrate more than 100 um into the tissue, thereby making this method not useful for transfecting the ventrally located MCNs in the hypothalamus *in vivo*. Therefore, we turned to the highly efficient recombinant AAV (AAV) method of gene transfer in vivo for further promoter deletion studies. This viral vector method had the additional virtue of being able to be stereotaxically targeted by microinjection to specific brain regions such as the SON, which had large numbers of OXT- and AVP MCNs in approximately equal numbers.

Viral vector methods are highly effective as a means to deliver genes into the nervous system in vivo [36], [37], [38], [39], [40] and have been used for gene transfer into hypothalamic neurons [40], [41], [42]. With respect to our study, we note that viral vector approaches have also been successfully used to study gene promoter domains that are involved in cell-type specific gene expression in vivo [43], [44], [45], [46], [47]. The experiments described in this paper use AAV vectors that contain promoter deletion constructs of the OXT gene promoter fused to EGFP reporters to transduce (transfect) neurons in the rat SON in vivo. AAV vectors have been specifically shown to be more efficient than lentiviral vectors in transducing neurons in the forebrain [48], [49].

After stereotaxic injection of the AAVs into rat SONs, we allow two weeks for expression of the EGFP, then perfuse fix the rat brains, and perform IHC on cryostat sections of the hypothalamus to assay the expression of the EGFP in either the OXT- or AVP MCNs. Injections of AAVs containing 568 bp of the DNA sequence upstream of the transcription start site (TSS) in the OXT gene are able to produce robust expression selectively in OXT- but not in the AVP –MCNs (Fig. 3). This was similar to the transgene which was shown in previous transgenic studies to produce cell-type specific expression in the MCNs [18]. Further experiments showed that AAV vectors containing 440 bp, 325 bp and 216 bp (but not 50 or 100 bp) upstream sequences of the OXT gene promoter can all support cell-type specific OXT gene expression in OXT MCNs and not in AVP MCNs (Fig. 4). It is noteworthy that the fluorescence shown in Fig. 4 for the p216, p100 and p50AAVs required amplification by EGFP antibody staining due to their lower levels of expression, whereas the intrinsic fluorescence from the p325 to p563 AAVs was sufficiently robust to be clearly observed without immunohistochemical amplification. For this reason we suggest there may be an enhancer located greater than 216 bases upstream of the OXT transcription start site. Fig. 4 also shows that the AAV containing the −50 bp and −100 bp upstream regions can produce EGFP expression in the SON, but non-selectively in the OXT-and AVP- MCNs, as might be expected of a “core promoter” region. Finally, we show in Fig. 5 that introns 1 and 2 and exons 2 and 3 in the OXT gene are not needed for cell-type specific expression in OXT MCNs. These findings further suggest that the AAV vector in vivo approach used here provide an effective general alternative to conventional transgenic approaches for the study of gene expression driven by promoter deletion constructs in the rat SON.

One surprising finding in this study is that the mechanism of the well-studied osmotic regulation of the OXT gene [6] is present in all of the deletion constructs studied, and appears to implicate the core promoter in the osmoregulation of the oxytocin gene. The images in Fig. 6 showing intrinsic fluorescence in SONs in control and salt loaded rats were captured with the same exposure times so that expression levels between dehydrated and salt loaded rats could be directly compared. This figure shows that the putative response elements associated with increased expression due to salt loading are present in the p100 and p50 promoter regions and thus are not linked with the cell-type specific expression domain between −216 to −100. The increase in expression of the EGFP in salt loaded rats is not accompanied by any changes in the specificity of cell-type expression (unpublished data, Figs. 3 and S3). One possibility is that the osmotic regulation resides near the core promoter domain, possibly at the Pol II binding site. The location of this regulatory element in the core promoter is consistent with the global changes in gene expression found in the SON in microarray studies [50], [51], and the changes in nuclear and somatic sizes of the MCNs in the SON [52], [53] during systemic, chronic osmotic perturbations. It is also possible that the non-canonical TATA (Goldberg-Hogness) box sequence, known to be present in the core promoters of the AVP and OXT genes [54], [55], might be involved in the osmotic regulation of the OXT gene. While it is possible that the non-canonical TATA box could be involved, we know of no paper in the literature that either shows evidence for or that suggests this possibility for the OXT gene. The only papers in the literature that address the osmotic regulation issue focus only on the AVP gene, and these are exclusively studies using heterologous cell lines (e.g, SCLC, HeLa, and HEK293). They report osmotic regulatory sites in the AVP gene that are located in the 5′ flanking sequence between −1500 and −532 bp upstream of the TSS [56], [57], and are clearly unrelated to our observations in the core promoter domain. The only evidence that the non-canonical TATA box could be involved in regulating the AVP gene has again been shown in heterologous cell lines [58], [59] and not in MCNs. Therefore, whether the non-canonical TATA box is involved in the osmotic regulation of the OXT and AVP genes remains completely speculative. However, in this regard, it would be interesting to know whether all the genes in the SON that have been shown in microarray studies to be osmoregulated in vivo [50] all contain non-canonical TATA boxes.

**Figure 7:**
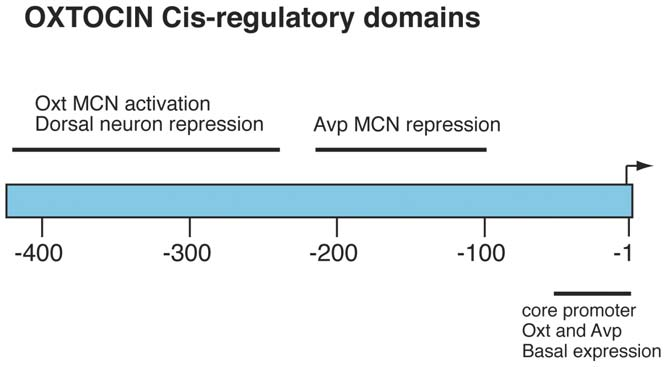
Diagram summarizing the cis-regulatory domains in the oxytocin gene promoter as suggested by the data in this study. The −50 bp and −100 bp OXT promoter domains are sufficient to produce expression of an EGFP reporter in both OXT and AVP MCNs in the SON, and in a population of smaller neurons just dorsal to the SON. The −100 to −216 bp domain appears to contain a repressor element that inhibits expression specifically in AVP MCNs. The −216 to −325 domain appears to repress expression in an unidentified dorsal neuron population above the SON, and the −216 to −440 bp domain may have enhancer elements which are operative in the OXT MCNs.

Based on data presented in this paper, we hypothesize that: 1) there is a repressor element, (RE) in the −216 to −100 5′ upstream region of the OXT gene that prevents its expression in AVP MCNs, 2) that there is another potential RE in the −325 to −216 region upstream of the TSS in the OXT gene which prevents expression in a non-MCN, unidentified dorsal neuron population above the SON, and 3) that there is an enhancer in the −216 bp to −440 bp domain region upstream of the TSS in the OXT gene that participates in the OXT MCN's expression (summarized in Fig. 7). In another series of experiments we injected the AAVs from p563 to p100 (illustrated in Fig. 1) into the rat cortex and found no detectable EGFP expression there (data not shown). Given these results, we propose that there is another repressor domain in the −100 to −1 region in the OXT promoter that prevents expression in extra-hypothalamic areas of the brain.

The most important finding in this paper with respect to regulation of cell-type specific expression in the SON is that the −216/−100 bp region in OXT gene promoter contains the key elements that determine its cell-type specific expression in the OXT MCNs in vivo, and this proposal is further informed by the extensive reports of in vitro studies that have focused on this promoter domain. Promoter deletion studies in the 1990s using a variety of heterologous cell lines identified this region in the OXT promoter as containing functional estrogen/retinoic acid receptor-like transcription factor binding sites. Richard and Zingg [60], [61] using 5′ deletion mutants of the human OXT gene that were expressed in Neuro 2A cells identified a −381 to +36 bp region in the OXT promoter that overlapped with an estrogen response element (ERE) motif between −164 to −152. Mutant constructs containing this region increased four-fold in expression in response to retinoic acid in cells that were co-transfected with a retinoic acid receptor α, thus suggesting that this domain in the OXT gene contained a retinoic acid receptor element (RARE). A similar domain was reported as being present in the bovine OXT gene promoter at −160/−152 bp, which was characterized as having an orphan chicken ovalbumin upstream promoter (COUP)-like element [62], [63] that bound a SF-1-like orphan receptor. Adan et al. [64], [65], [66] reported the presence of a core domain at 172/−148 in the rat gene promoter that increased OXT gene expression in P19 cells in response to thyroid hormone (T3). These authors subsequently referred to this domain as the Composite Hormone Response Element (CHRE) since it could bind a variety of classical and orphan nuclear hormone receptors (see Burbach, [67], for a review of the CHRE concept), and Lopes da Silva and Burbach [68], for a discussion of the diversity of nuclear hormone receptor super family members found in brain. Most of the above nuclear hormone receptors (NHRs) that were found to activate OXT-promoter gene expression in heterologous cell lines, were classical NHRs. However, Chu and Zingg [69] showed that a mouse OXT gene promoter transfected into Neuro 2A is activated by ROR α, a member of the ROR/RZR orphan receptor subfamily, to produce a six-fold increase in transcription at two sites (−160 bp and 180 bp) in the CHRE region.

While many studies identified NHRs that could activate OXT gene expression, there are also reports that describe repressor actions of NHRs in the same CHRE domain. The earliest example of this was the demonstration of a negative retinoic acid element (termed nRARE) at −178/−158 bp in a rat OXT gene promoter transfected into an African green monkey kidney cell line [70]. Other examples, are the demonstration that SF-1 binding at the −160 bp element in the bovine OXT promoter inhibited the activation of OXT gene expression induced by COUP-TF binding [71], and a report that estrogen, retinoic and, or thyroid hormone stimulation of OXT gene expression in P19 cells was fully inhibited by COUP-TF-1 [72]. Along the same lines, another group reported that COUP-TF II and Ear 2 act as silencers of estrogen stimulated transcription of human OXT gene expression in Neuro 2A cells [73]. Another NHR present in the SON is estrogen receptor beta, and interestingly its expression is normally greater in AVP MCNs than in OXT MCNs and is inversely correlated with the level of expression of the neuropeptide genes [74]. This finding suggests a possibility that estrogen receptor beta might play a role to repress OXT gene expression in the AVP MCNs.

The above in vitro studies identified specific binding sites within the −216/−100 bp domain in the OXT gene promoter that we have shown in our studies are responsible for the cell-type specific expression of the OXT gene in MCNs in the SON in vivo. Since the in vitro studies have shown that specific NHRs can act as either activators or inhibitors of OXT gene expression, these data are consistent with our hypothesis that elements in this 116 bp domain could regulate the activation or repression of OXT gene transcription in the OXT- or AVP MCNs, respectively, depending on the specific NHRs being expressed in each of these cell-types. In an effort, to identify the specific NHRs that are expressed in the SON, Lopes de Silva and Burbach [75]used a polymerase chain reaction/homology cloning strategy to identify NHRs that are present in the microdissected SON. They found that five classical receptors and four orphan receptors were expressed in the SON, but only one of these, the thyroid hormone receptor-α (THRα) was found by in situ hybridization histochemistry to be expressed in the MCNs [75], [76]. Unfortunately, there were no experiments reported that provide information as to whether the THRα is preferentially expressed in the OXT- or the AVP MCNs. Experiments are currently in progress in our laboratory to further dissect the −216/100 bp domain in vivo, to determine whether there is a selective OXT-versus AVP-MNC expression of THRα and other candidate NHRs.

## Supporting Information

Figure S1 **Shows a low power view of a coronal section of the rat hypothalamus after injection into the SON of an AAV containing the pan-specific CMV promoter fused to an EGFP reporter.** Note that the SON shows intense EGFP fluorescence and that areas dorsal and medial(towards the 3 V) also show significant although less dense cellular fluorescence indicating the wide area of potential transduction deriving from this AAV injection. Dotted line shows dorsal boundary of the SON. Abbreviations: OC, optic chiasm; 3 V, third ventricle. Scale line is 150 µm.
(TIF)

Figure S2 **Illustrates the results from an experiment where the EGFP fluorescence shown in the SON in A, two weeks after injection of a CMV-EGFP-containing AAV vector, is compared to the immunofluorescence observed after immunostaining the same section with the PS 45 antibody, which detects both AVP- and OXT-associated neurophysins (in B), and therefore identifies both the AVP- and OXT- MCNs in the SON.** The merged view is shown in C. Measurements of the numbers of MCNs in the SON that colocalize the PS 45-ir with the EGFP fluorescence forms the basis for the determination of the AAV efficiency of transduction in the SON (see Methods and text). Abbreviations: OC, optic chiasm. Scale line is 100 µm.
(TIF)

Figure S3 **A and D show the detection of EGFP immunofluorescence (using an antibody against EGFP) in the SON after injection of a p563OXT-EGFP-containing AAV vector and with the use of the salt loading paradigm (see Methods).** The section in A was also immunostained with the PS 41 (AVP-neurophysin-specific) antibody shown in B, and the merge of A and B is shown in C. Similarly, The section in D was also immunostained with the PS 38 (OXT-neurophysin-specific) antibody shown in E, and the merge of D and E is shown in F. Note that the use of EGFP antibody to amplify the EGFP detection and the use of salt loading to increase the expression of the p563 construct (see Fig. 6) did not alter its specificity of expression (compare these data with the data in Fig. 3).Abbreviations: OC,optic chiasm. Scale line is 100 µm.
(TIF)
