# Correction: Cell-Type Specific Oxytocin Gene Expression from AAV Delivered Promoter Deletion Constructs into the Rat Supraoptic Nucleus in vivo

**DOI:** 10.1371/annotation/2d183615-8b34-4ea4-ae9b-9833d6079d11

**Published:** 2012-06-11

**Authors:** Raymond L. Fields, Todd A. Ponzio, Makoto Kawasaki, Harold Gainer

Figure S3 is a duplicate of Figure S2. Please view the correct Figure S3 here: 

[^] 

