# Supplementary figures and images for: Cell-Type Specific Oxytocin Gene Expression from AAV Delivered Promoter Deletion Constructs into the Rat Supraoptic Nucleus in vivo
Source: PLoS One. 2012 Feb 21;7(2):e32085. doi: 10.1371/journal.pone.0032085 (PMC3283729; doi:10.1371/journal.pone.0032085)

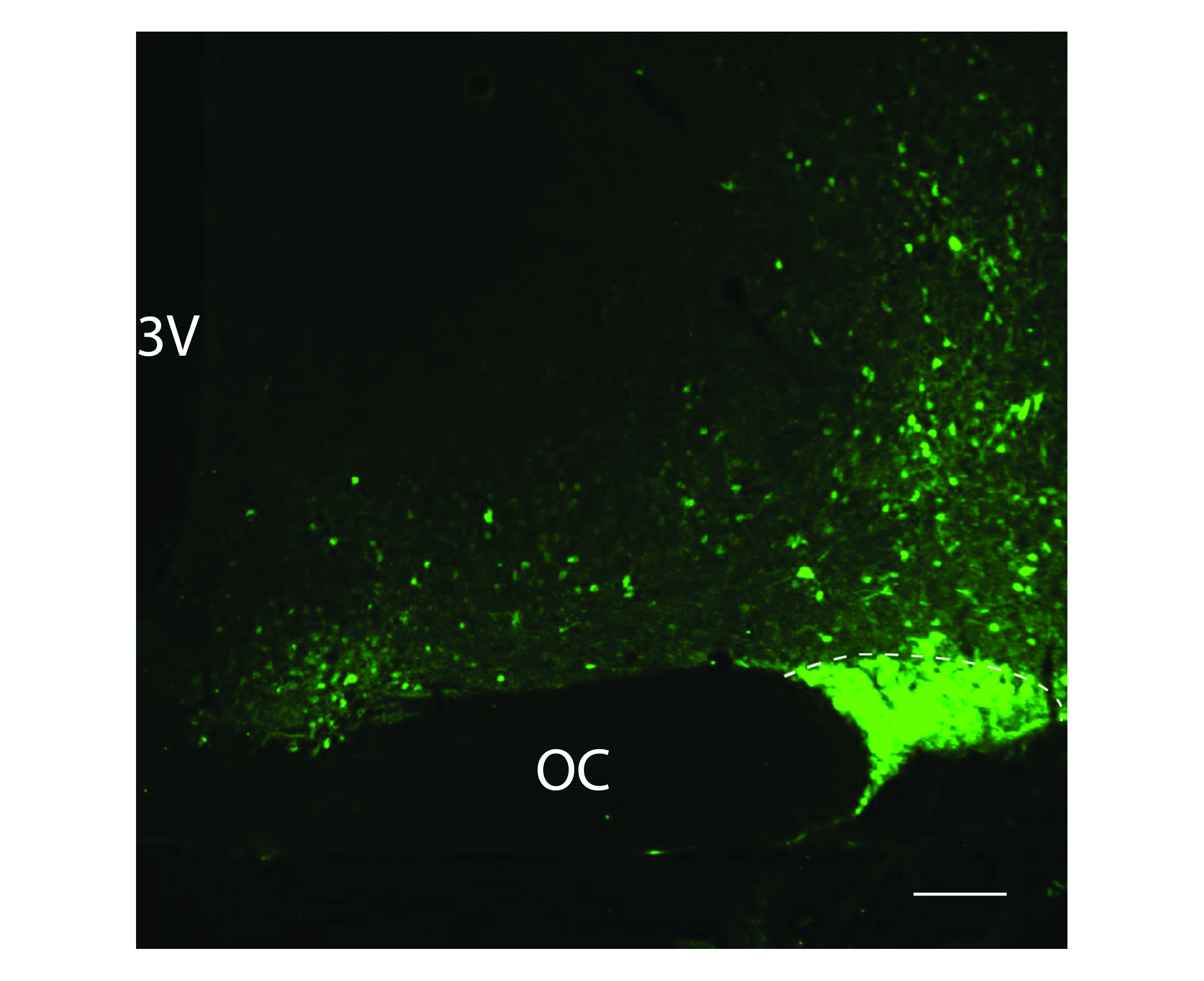

Supplement: Figure S1 — Shows a low power view of a coronal section of the rat hypothalamus after injection into the SON of an AAV containing the pan-specific CMV promoter fused to an EGFP reporter. Note that the SON shows intense EGFP fluorescence and that areas dorsal and medial(towards the 3 V) also show significant although less dense cellular fluorescence indicating the wide area of potential transduction deriving from this AAV injection. Dotted line shows dorsal boundary of the SON. Abbreviations: OC, optic chiasm; 3 V, third ventricle. Scale line is 150 µm. (TIF) [file pone.0032085.s001.tif]

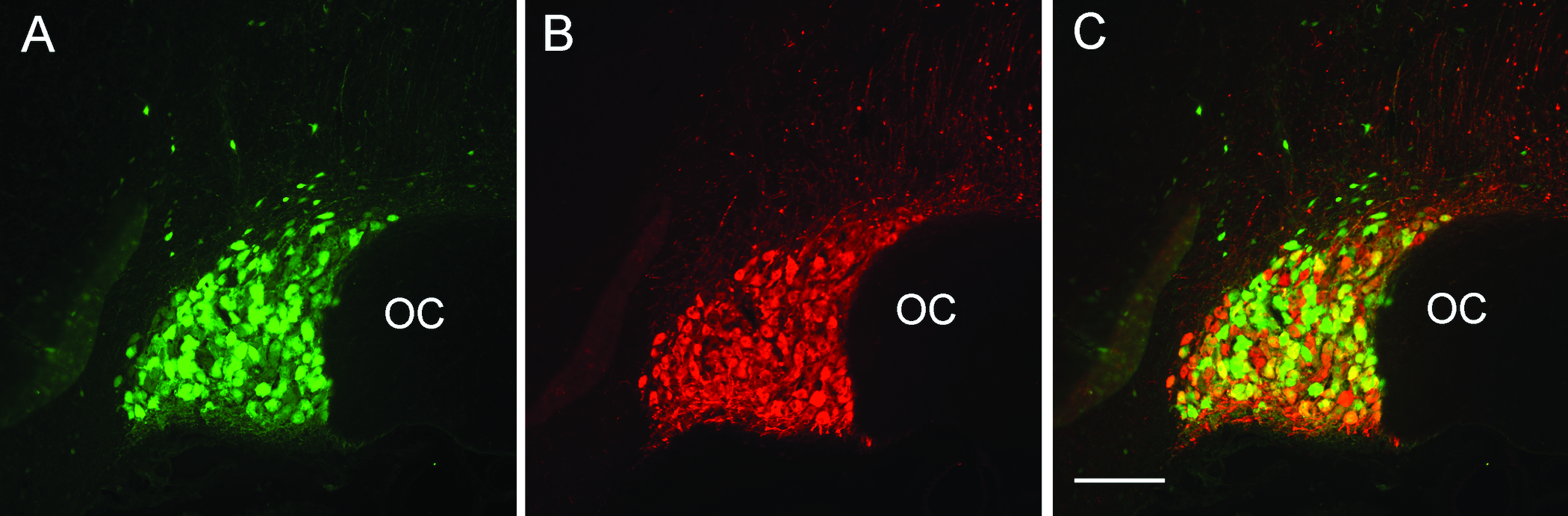

Supplement: Figure S2 — Illustrates the results from an experiment where the EGFP fluorescence shown in the SON in A, two weeks after injection of a CMV-EGFP-containing AAV vector, is compared to the immunofluorescence observed after immunostaining the same section with the PS 45 antibody, which detects both AVP- and OXT-associated neurophysins (in B), and therefore identifies both the AVP- and OXT- MCNs in the SON. The merged view is shown in C. Measurements of the numbers of MCNs in the SON that colocalize the PS 45-ir with the EGFP fluorescence forms the basis for the determination of the AAV efficiency of transduction in the SON (see Methods and text). Abbreviations: OC, optic chiasm. Scale line is 100 µm. (TIF) [file pone.0032085.s002.tif]

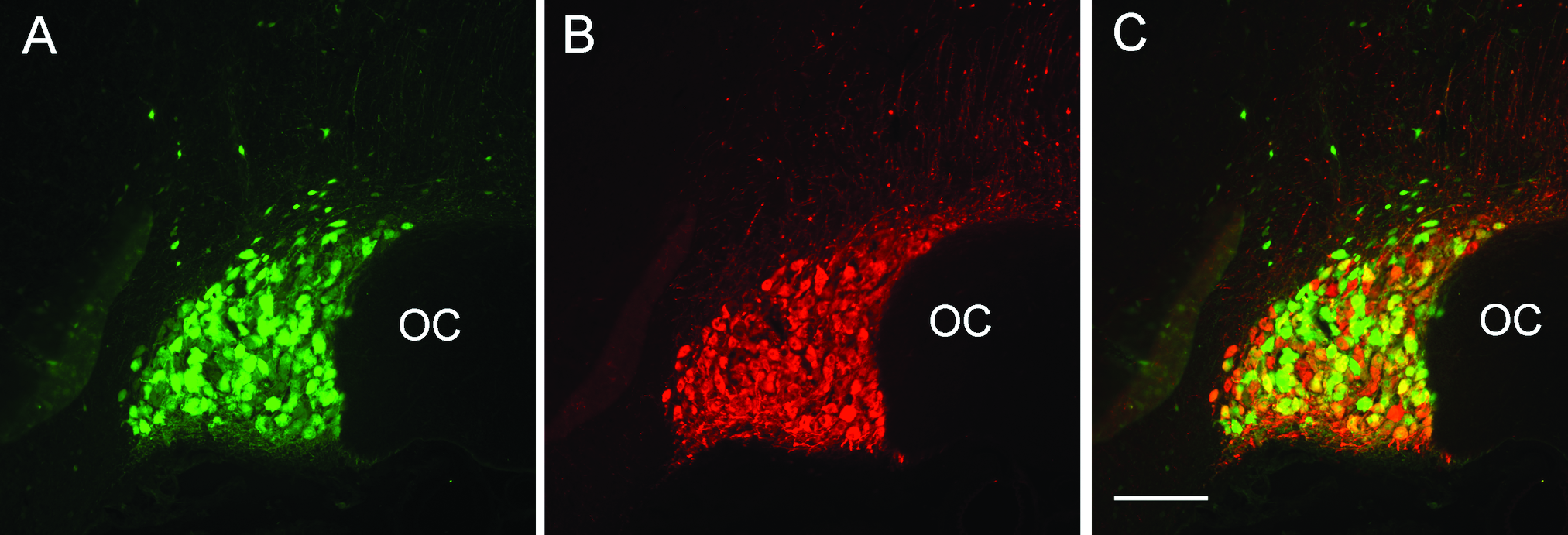

Supplement: Figure S3 — A and D show the detection of EGFP immunofluorescence (using an antibody against EGFP) in the SON after injection of a p563OXT-EGFP-containing AAV vector and with the use of the salt loading paradigm (see Methods). The section in A was also immunostained with the PS 41 (AVP-neurophysin-specific) antibody shown in B, and the merge of A and B is shown in C. Similarly, The section in D was also immunostained with the PS 38 (OXT-neurophysin-specific) antibody shown in E, and the merge of D and E is shown in F. Note that the use of EGFP antibody to amplify the EGFP detection and the use of salt loading to increase the expression of the p563 construct (see Fig. 6) did not alter its specificity of expression (compare these data with the data in Fig. 3).Abbreviations: OC,optic chiasm. Scale line is 100 µm. (TIF) [file pone.0032085.s003.tif]
