# Supplementary figures and images for: Correction: Cell-Type Specific Oxytocin Gene Expression from AAV Delivered Promoter Deletion Constructs into the Rat Supraoptic Nucleus in vivo
Source: PLoS One. 2012 Jun 11;7(6):10.1371/annotation/2d183615-8b34-4ea4-ae9b-9833d6079d11. doi: 10.1371/annotation/2d183615-8b34-4ea4-ae9b-9833d6079d11 (PMC3378652; doi:10.1371/annotation/2d183615-8b34-4ea4-ae9b-9833d6079d11)

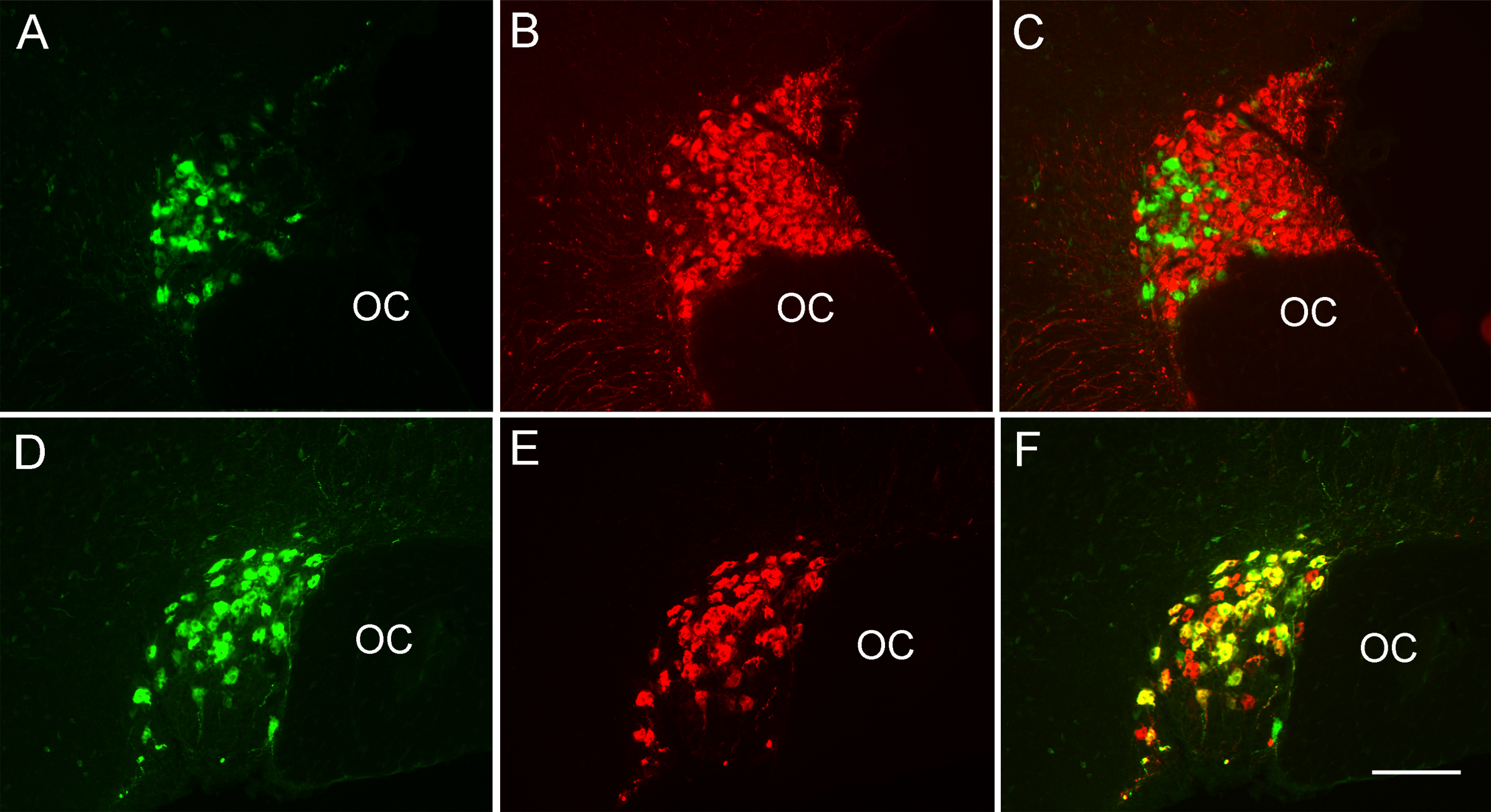

Supplement: Supplementary file 1 [file pone.2d183615-8b34-4ea4-ae9b-9833d6079d11.s001.tif]
